# Costing conservation: an expert appraisal of the pollinator habitat benefits of England’s entry level stewardship

**DOI:** 10.1007/s10531-014-0660-3

**Published:** 2014-03-08

**Authors:** T. D. Breeze, A. P. Bailey, K. G. Balcombe, S. G. Potts

**Affiliations:** Centre for Agri-Environmental Research, University of Reading, Reading, UK

**Keywords:** Agri-environment schemes, Pollination services, Entry level stewardship

## Abstract

Pollination services provided by insects play a key role in English crop production and wider ecology. Despite growing evidence of the negative effects of habitat loss on pollinator populations, limited policy support is available to reverse this pressure. One measure that may provide beneficial habitat to pollinators is England’s entry level stewardship agri-environment scheme. This study uses a novel expert survey to develop weights for a range of models which adjust the balance of Entry Level Stewardship options within the current area of spending. The annual costs of establishing and maintaining these option compositions were estimated at £59.3–£12.4 M above current expenditure. Although this produced substantial reduction in private cost:benefit ratios, the benefits of the scheme to pollinator habitat rose by 7–140 %; significantly increasing the public cost:benefit ratio. This study demonstrates that the scheme has significant untapped potential to provide good quality habitat for pollinators across England, even within existing expenditure. The findings should open debate on the costs and benefits of specific entry level stewardship management options and how these can be enhanced to benefit both participants and biodiversity more equitably.

## Introduction

Pollination is a key ecosystem service, underpinning the reproduction of ~78 % of temperate flowering plants (Ollerton et al. 2011) and influencing yields of ~75 % of global crops (Klein et al. 2007). Within the UK, insect pollination services to crops increase market output by £430 M as of 2007, equivalent to ~8 % of total crop value (Smith et al. 2011). Despite this contribution to crop agriculture, substantial declines in wild and managed pollinators have been observed across the UK (Carvalheiro et al. 2013; Potts et al. 2010) due to a combination of climate change, pesticide exposure, disease and the loss of good quality habitat (Vanbergen 2013). While managed honeybees can provide pollination services to a wide range of crops (Klein et al. 2007), their contribution to actual service delivery is often small compared with wild bees (Garibaldi et al. 2013). Loss of good quality habitat has primarily been driven by long-term agricultural intensification, with diverse crop landscapes being replaced with expansive monocultures at the expense of semi-natural habitats and boundary features (Burgess and Morris 2009). Intensified agriculture is further characterised by high agrochemical inputs and livestock herd density; increasing exposure to potentially harmful insecticides (e.g. Gill et al. 2012; Henry et al. 2012) and reducing the diversity of flowering plants through herbicide and fertiliser application and overgrazing (Isbell et al. 2013; Carvalheiro et al. 2013).

Within the EU, agricultural intensification has been widely encouraged by the common agricultural policy (CAP) which offered production linked subsidies to farmers in exchange for price controls (Stoate et al. 2009). Reforms to CAP in 2005 continued the decoupling of subsidies from production and relaxed price controls, increasing market influence on prices paid to producers. However, despite these reduced incentives to maximise production, grazing intensity and fertiliser consumption remain similar to prior levels (DEFRA 2013). Later reforms also removed requirements for claimants to leave part of their land in low or no production (“set-aside”), much of which was managed as potentially beneficial semi-natural habitat (Dicks et al. 2010). Consequently, there remains a need to actively mitigate the impacts of agriculture by restoring habitat quality and connectivity to secure pollination service supply (Hatfield and LeBuhn 2007).

The principal means of providing habitat for pollinators within the farmed landscape are agri-environment schemes (AES), part of CAP’s second pillar of funding, which pays land owners for their uptake of biodiversity and other measures on their land. Although there are several AES within the UK, the most widespread is England’s entry level stewardship (ELS), which covers ~62 % of English farmland (5.7 M Ha) as of January 2013 (Natural England 2013a). This scheme is a key component of the current government’s plan to produce a sustainable ecological network by acting as corridors between primary source habitats (DEFRA 2011). ELS agreements are short-term, lasting 5 years, and allow farmers to select from and combine a broad range of management options to meet their requirements. Both of these attributes are considered desirable by farmers (Sutherland 2009) and have likely driven the widespread uptake of the scheme. More specialised variants of ELS are available for organic farming and severely disadvantaged areas in the uplands. Although management measures in ELS have been demonstrated to benefit pollinators, such as nectar flower mixes and low input pastureland (Scheper et al. 2013), payments for ELS are fixed regardless of the combination of options used to qualify and therefore uptake has typically been biased towards lower cost, often opportunistic options (e.g. low frequency hedge cutting), that are thought to be less beneficial to biodiversity (Sutherland 2009; Hodge and Reader 2010). Furthermore, much of this uptake has been in low productivity areas where AES are thought to be less beneficial due to high existing habitat diversity (Hodge and Reader 2010; Scheper et al. 2013; Cloither 2013). Like all AES, the monitoring of ELS is limited by its budget, allowing for potentially high levels of poor or false implementation (Kleijn and Sutherland 2003) and can vary strongly in their effectiveness between scheme designs (Kleijn et al. 2006).

Recently accepted reforms to CAP include a greening requirement in order to claim the full value of subsidies. This includes a mandatory 5 % of land to be designated as ecological focus areas, comprised of a combination of hedges, trees, fallow land, grassland maintenance and low input margins (European Commission 2013). Although this may result in ELS being replaced or radically overhauled, there is still a need to appraise benefits of the current management options under the scheme in order to better inform potential successors. Whilst evidence exists to suggest ELS options can improve the quality of insect pollinator habitats (Kleijn et al. 2006; Potts et al. 2009; Pywell et al. 2011), the benefits of most options remain unknown, and are likely to remain so given the significant investment and time in conducting robust empirical studies. Furthermore, although economic valuations of pollination services have been used to justify expenditure on mitigation efforts, to date only one study has compared these benefits to any costs of conservation actions (Cook et al. 2007). The purpose of this study is therefore twofold; first, to provide a simple appraisal of the relative benefits of all ELS options to providing good quality pollinator habitat. Secondly this study provides an estimate of the cost in adapting the currently utilised ELS area towards pollinator conservation provision by redistributing the current national mix of ELS options towards one reflective of the relative benefits to insect pollinator habitat.

## Methods

This study focuses upon the entry level stewardship (ELS) as it is both very widespread, incorporating 5 M ha of English Farmland (Natural England 2013a), and has many options that are applicable to other UK and European agri-environment schemes (AES). ELS allows enrolled participants to select from a suite of management options, each with a point value. Participants must select 30 points worth of options per hectare of their enrolled holding and are paid £30 per hectare in return. These payments total £163 M per annum as of January 2013 with a further £1.4 M spent on monitoring (Natural England 2013a). Due to the timing of the expert survey, this study focuses upon the third edition of ELS (Natural England 2010), although a fourth edition is now in use (Natural England 2013b).

### Estimating habitat benefits

To evaluate the potential benefits of each option for providing good quality habitat for pollinators, an expert panel survey was conducted. As primary ecological data on the responses of pollinators to ELS management options is limited to a few options and focal pollinator taxa (e.g. Potts et al. 2009; Pywell et al. 2011; Carvell et al. 2007), an expert panel was used to evaluate the relative benefits of each ELS option to pollinator habitat. Similar methods have been used to assess pressures (Kuldna et al. 2009) and model habitat suitability (Lonsdorf et al. 2009) for pollinators. Experts were academics with at least three publications on pollinator ecology and non-academics recommended on the basis of 10 or more years’ experience in UK bee or hoverfly ecology. In total 35 experts were approached in March 2010. Delphi panel and Bayesian models (Czmebor et al. 2011) were considered but not pursued due to the difficulty in eliciting multiple responses and limited primary data available for modelling outcomes.

Experts were surveyed via e-mail, following a small pilot survey, with reminders sent to non-respondents after 2 and 4 weeks. Respondents were asked to rate each option on providing good quality habitat (i.e. suitable nesting or forage resources) for a wide range of wild pollinators (bees and hoverflies) in farmed landscapes across the UK on a scale from 0 (no benefit) to 3 (great benefit). This simple scale was selected due to the volume of options under consideration potentially increasing respondent fatigue. Experts were also asked to report their confidence in their response on a four point scale from (0) not confident to (3) very confident. From this the Pollinator Habitat Benefit (PHB) values, weighted by expert confidence, of each option were calculated as:

1 $$PHB_{i} = \frac{{\sum_{e = 1}^{E} {(H_{ei} } \times C_{e} )}}{{\sum\nolimits_{e = 1}^{E} {C_{e} } }}$$

where *H*_*ei*_ is the habitat quality score allocated by expert e to option i and *C*_*e*_ is expert’s self-reported confidence. To avoid respondent fatigue, only one confidence measure was taken for all options. To control for the effects of between expert variation (Czmebor et al. 2011) this was then divided by the total confidence values to produce an average across all experts within the original 0–3 scale.

### Redistributing ELS options

Using the expert PHB weights, a series of models were developed to redistribute the 2012 composition of ELS options in a manner which reflected their relative benefits for providing habitat for insect pollinators. These models allocated units of each option based upon the benefit they provided to pollinator habitats relative to other options within specific categories; with the most beneficial option allocated the greatest number of units and the least beneficial allocated the least units. This method was chosen over optimisation models for the sake of methodological simplicity, particularly given the high number of variables involved, and to avoid scenarios dominated by high benefit and/or low cost options. The changes in costs and habitat benefit (measured as the sum value of PHB) were then appraised for each model. The number of units and total ELS points generated by each option as of December 2012 were obtained from Natural England databases (Cloither 2013, Pers Comm) excluding options that are no longer available (e.g. EM1-4) or those that relate only to historic or built features (e.g. ED1-5) and water bodies. Mixed stocking (EK5) was also excluded to avoid double counting as this option can be combined with other grassland options. Options relating to severely disadvantaged areas (EL1-6) and ELS variants, (organic and upland ELS), were not included to reduce respondent fatigue and maintain model simplicity by only considering broadly applicable options.

The remaining options were grouped into categories based upon their management units (hedge/ditch options, managed in metres/hectares; further subdivided into grassland and arable, and plots/trees) and the area and points values of options within each category were summed to produce a baseline estimate (Table 1). For option EC4, which could be present in both grassland and cropland, the area and points were distributed proportionate to the relative area of the two groups; 24 % cropland and 76 % grassland (DEFRA 2013).

**Table 1:** Baseline data

	Units	Points
Total length (H)	191,556,761 m	48,503,029
Total arable area (A)	133,123 ha	37,178,883
Total grassland area (G)	420,225 ha	45,219,223
Total trees and plots (P)	206,993	2,254,303
Total 2012		133,155,438

*Key* *Units* the number of units of each option category in the baseline mix considered. Points: The total ELS points of all units of the options considered

**Table 2:** Weighted and unweighted mean PHB scores attributed to 2010 ELS options

ELS option	Description	Type	2012 Pts %	PHB	WPHB
EB1/2	Hedgerow management for landscape	H	17.5	1.83	1.83
EB3	Enhanced hedgerow management	H	8.8	1.94	1.96
EB6	Ditch/half ditch management	H	3.2	1.33	1.38
EB7	Half ditch management	H	0.5	1.33	1.40
EB8/9	Combined hedge and ditch management (inc EB1/2)	H	3.6	1.83	1.88
EB10	Combined hedge and ditch management (Inc EB3)	H	1.9	1.94	2.00
EB12/13	Earth bank management	H	0.6	1.61	1.60
EC1	Protection of in-field trees (arable)	T	0.3	0.94	1.00
EC2	Protection of in-field trees (grassland)	T	1.3	1.00	1.04
EC3	Maintenance of woodland fences	H	0.2	0.72	0.69
EC4	Management of woodland edges	A/G	0.4	1.89	1.88
EC23	Establishment of hedgerow trees by tagging	T	<0.1	0.89	0.90
EC24	Hedgerow tree buffer strips on cultivated land	A	<0.1	1.78	1.81
EC25	Hedgerow tree buffer strips on grassland	G	<0.1	1.78	1.81
EE1/2	2/4 m buffer strips on cultivated land	A	3	1.50	1.54
EE3	6 m buffer strips on cultivated land	A	6	1.44	1.50
EE4/5/6	2/4/6 m buffer strips on intensive grassland	G	0.7	1.44	1.50
EF1	Field corner management	A	7.3	1.67	1.75
EF2/3	Wild bird seed mixture	A	2.7	1.50	1.65
EF4/5	Nectar flower mixture	A	1.2	2.83	2.83
EF6	Over-wintered stubbles	A	5	0.44	0.44
EF7	Beetle banks	A	0.1	1.17	1.13
EF8	Skylark plots	T	0.1	0.61	0.63
EF9	Cereal headlands for birds	A	<0.1	0.83	0.83
EF10	Unharvested cereal headlands for birds & rare plants	A	<0.1	0.89	0.96
EF11	Uncropped, cultivated margins for rare plants	A	0.1	1.78	1.81
EF13	Uncropped cultivated areas for ground-nesting birds	A	0.1	1.17	1.17
EF15	Reduced herbicide cereal crop preceding over-wintered stubble	A	0.1	0.61	0.60
EF22	Extended overwintered stubbles	A	1.6	0.50	0.50
EG1	Under sown spring cereals	A	0.4	0.51	0.54
EG4	Cereals for whole crop silage followed by over-wintered stubbles	A	0.1	0.33	0.33
EK1	Take field corners out of management	G	0.2	1.39	1.40
EK2	Permanent grassland with low inputs	G	18.4	1.33	1.31
EK3	Permanent grassland with very low inputs	G	13.8	1.72	1.77
EK4	Manage rush pastures	G	0.5	0.67	0.63

*Key* *2012 Pts* the % of total ELS points (among the options considered) accounted for by the option(s) in 2012, *Type* option category, *H* Hedge/ditch, *A* arable, *G* grassland, *P* plot/tree, *PHB* the unweighted mean PHB values from all 18 experts, *WPHB* the mean PHB values of all 18 experts following weighting

**Table 3:** Number of units of each ELS option after redistribution

ELS option	Type	Baseline	Model A		Model B		Model C	
		Units	Units	% change	Units	% change	Units	% change
EB1/2	H	106.1 M	17.9 M	−83	25.0 M	−76	20.3 M	−81
EB3	H	27.9 M	44.3 M	59	26.7 M	−4	21.7 M	−22
EB6	H	17.8 M	17.8 M	<1	18.7 M	5	15.3 M	−14
EB7	H	9.1 M	6.0 M	−34	19.0 M	110	15.5 M	71
EB8/9	H	11.5 M	34.8 M	202	25.6 M	122	20.8 M	81
EB10	H	4.6 M	60.3 M	1,221	27.3 M	497	22.2 M	386
EB12/13	H	7.3 M	9.1 M	24	21.9 M	200	17.8 M	144
EC1	T	28,005	105,209	276	71,613	156	110,965	296
EC2	T	154,668	75,345	−51	74,596	−52	115,589	−25
EC3	H	7.4 M	1.5 M	41	9.4 M	34	7.6 M	4
EC4 (Arable)	A	297	11,707	2,2042	8,604	2,794	13,871	4,566
EC4 (Grass)	G	941	98,530	21,9235	27,901	2,864	13,871	1,373
EC23	T	4,181	5,891	−80	64,153	1,434	99,406	2,278
EC24	A	54	11,912	3,838	8,317	15,359	13,408	24,822
EC25	G	46	100,258	10,366	26,971	58,905	13,408	29,233
EE1/2	A	10,717	9,720	−9	7,074	−34	11,405	6
EE3	A	19,814	9,858	−50	6,883	−65	11,097	−44
EE4/5/6	G	2,285	79,102	3,326	22,321	887	11,097	386
EF1	A	24,429	11,502	−53	8,030	−67	12,946	−47
EF2	A	8,037	12,169	51	7,552	−6	12,175	51
EF4	A	3,614	20,949	480	13,002	260	20,960	480
EF6	A	55,814	863	−98	2,008	−96	3,236	−94
EF7	A	125	10,712	8,477	5,162	4030	8,322	6,558
EF8	T	20,139	20,549	2	44,758	122	69,353	244
EF9	A	403	1,369	240	3,824	849	6,165	1,430
EF10	A	188	5,196	2,664	4,398	2239	7,089	3,671
EF11	A	319	11,912	3,634	8,317	2507	13,408	4,103
EF13	A	338	6,901	1,679	5,354	1280	8,631	2,124
EF15	A	670	1,936	189	2,772	314	4,469	567
EF22	A	5,274	3,368	−36	2,294	−57	3,699	−30
EG1	A	2,379	1,780	−25	2,486	4	4,007	68
EG4	A	563	1,260	124	1,530	172	2,466	338
EK1	G	543	77,210	14,119	20,771	3,725	10,326	1,802
EK2	G	289,017	15,428	−95	19,531	−93	9,709	−97
EK3	G	122,567	36,733	−70	26,531	−79	13,100	−89
EK4	G	4,827	12,964	169	9,300	93	4,624	−4

**Table 4:** Total private and public cost:benefit changes under the three ELS redistribution models

Model	Costs (£M)	ELS Credits (£M)	Private C:B (£)	Cost/ha (£)	HQ Benefits (M)	Public C:B (£)
Baseline	32.2	133.2	1:4.13	7.3	200 M	1:1.50
Model A	91.4 (+286 %)	277.5 (+108 %)	1:3.04 (−27 %)	10 (+37 %)	480 M (+140 %)	1:1.73 (+15 %)
Model B	48.8 (+52 %)	133.2	1:2.73 (−34 %)	11.1 (+52 %)	228 M (+14 %)	1:1.72 (+15 %)
Model C	44.6 (+39 %)	133.2	1:2.98 (−28 %)	10.1 (+37 %)	214 M (+7 %)	1:1.61 (+7 %)

*Key* *Costs* Total annual cost to land-owners of the mix of ELS options generated. ELS Credits: the total ELS credit value. Private C:B: the relative benefits to farmers, in terms of ELS payments, per £1 of cost in establishing and maintaining the option mix generated. Cost/ha: average annual costs per hectare enrolled in the scheme (ELS credits/30). HQ Benefits: The sum value of pollinator habitat quality arising from the combination of options from the model. ELS Credits: the total ELS credit value of the option mix generated. Public C:B: the relative public benefits in terms of HQ, per £1 of ELS credits spent.  % changes relative to the baseline are presented in *brackets*

**Table 5:** Total units of each option type under the three ELS redistribution models

Model	Hedge/ditch options (Mm)	Grassland options (ha)	Arable options (ha)	Tree/plot options (no.)
Baseline	191.6	420,225	133,123	206,933
Model A	191.6	420,225	133,123	206,933
Model B	164.4 (−11 %)	153,147 (−64 %)	97,608 (−27 %)	216,738 (+23 %)
Model C	138.8 (−39 %)	61,656 (−85 %)	154,670 (+16 %)	388,569 (+88 %)

*Key* *Length options* total length of all length based options, *Grassland options* total area of all grassland area based options, *Arable* *options* total area of all arable area based options, *Tree/plot options* total numbers of tree and plot based options.  % changes relative to the baseline are presented in brackets

**Table 6:** Changes in total costs to producers and total pollinator habitat quality benefits under the three sensitivity analyses

	Model A		Model B		Model C	
	Total costs	PHQ	Total costs	PHQ	Total costs	PHQ
Sensitivity 1	+£1,480 (<1 %)	+316 (< 1 %)	−£1,419 (< 1 %)	−65,870 (< 1 %)	−£0.2 M (< 1 %)	−0.26 M (1.2 %)
Sensitivity 2	−£0.2 M (<1 %)	−357,145 (< 1 %)	−£4,403 (< 1 %)	−2.5 M (−1.1 %)	−£0.9 M (2 %)	−3 M (−1.4 %)
Sensitivity 3	−£8.3 M (9 %)	−85 M (−31 %)	+£0.3 M (< 1 %)	−95 M (−41 %)	−£4.1 M (9 %)	−87 M (−40 %)

*Key* All values are relative to their respective results in Table 2, relative percentage changes are indicated in brackets. *Sensitivity 1* Jackknifed sample removing individual experts (average of all jackknives presented), *Sensitivity 2* PHB unweighted by expert confidence, *Sensitivity 3* PHB unweighted by expert opinion

For each option a habitat quality (HQ) score was calculated as:

2 $$HQ_{i} = PHB_{i} \times ELS_{i}$$

where *ELS*_*i*_ is the ELS points value (and therefore farmer payment) attached to each unit of option i. This weights the quantitative metric of option quality relative to the scale of their implementation as a single hectare of habitat will typically provide a substantially greater total resource than a single metre of habitat. How ELS points are derived is presently unclear as although EU rules state they must be based upon their costs, including income foregone, earlier and recent revisions taking into account the biodiversity benefits of options have moved away from this initial approach (Natural England 2012, 2013b). As such ELS points largely represent relative general biodiversity benefit, which is then weighted by the expert PHB scores. To give a measure of the value of each option relative to all other options with the same unit category (*c*), proportional habitat quality (*pHQ*_*ic*_) values are then estimated as:

3 $$pHQ_{ic} = \frac{{HQ_{ic} }}{{\mathop \sum \nolimits_{i = 1}^{C} HQ_{ic} }}$$

The *pHQ* score for option i therefore represents its benefit to pollinator habitat relative to all other options within category *c*. *pHQi* scores are therefore always between 0 and 1 and the sum of all *pHQi* scores for a given category of c always equal 1. Using these *pHQ* values, three variant analyses were conducted to redistribute the overall composition of options towards a composition which reflects the relative benefits of the options for providing good quality habitat for pollinators. Model A generates a mix of options that redistribute the absolute area of ELS options currently utilised to reflect their relative benefits to pollinator oriented habitat. It thus redistributes the composition of options based upon the total utilised area of options within each category (i.e. the most beneficial option will take up the greatest number of units and so on). The area of different option categories is maintained to reflect current uptake patterns and preferences. This model allows the total number of ELS points, and therefore the total area of English farmland enrolled in the scheme, to expand, however no additional area of land is taken out of production.

$$U_{ic} = \mathop \sum \nolimits U_{c} \times pHQ_{ic}$$

where *U*_*ic*_ is the redistributed number of units of option *i* in category *c*, *Uc* is the total number of units (meters, hectares or trees/plots) in the category and *pHQ*_*ic*_ is the percentage of total *HQ* (calculated as in Eq. 2) in each option represents within the category. As such each option is allocated a percentage of the total units of category c based upon their relative benefit to pollinator habitat.

Model B generates a mix of options that maintains the current ELS budget, allowing the absolute area of options within the four categories to change. This is accomplished by redistributing the percentage of total ELS points in each option category based upon their pHQ scores (i.e. the most beneficial option will account for the greatest number of points within the category and so on). The number of units of each option is then the total points divided by the options ELS points value. Again, expenditure on categories is maintained to better reflect current enrolment and preferences. This allows the absolute area covered by ELS options to vary, however the total area enrolled in ELS, and the subsequent taxpayer payments, will remain the same.

$$P_{ic} = \mathop \sum \nolimits P_{c} \times pHQ_{ic}$$

where *P*_*ic*_ is the total ELS points accounted by option *i* in category *c*, *P*_*c*_ is the total ELS points produced by options in category c.

Model C also maintains current ELS budget, however, under this model the ELS points of all options are pooled regardless of their category and the redistribution is based upon the habitat quality benefits of each option in relation to all other options, regardless of their category. As such the most beneficial of all available options will represent the greatest percentage of total redistributed ELS points and so on. As with model B, this allows the number of units of each option to change, although now there is a degree of substitution between option categories and which may affect their prevalence in the overall ELS. To prevent the outputs of this model from being dominated by arable and grassland options, many of which are worth several hundred ELS points, the ELS points for hedge/ditch and plot/tree based options were multiplied by 1,000 (assuming 1 m^2^/unit of hedge/ditch options) and 10 (assuming 100 m^2^/unit of plot options) respectively to scale points of these options relative to 1 ha.

$$T_{i} = \mathop \sum \nolimits T \times tHQ_{i}$$

*T*_*i*_ represents the ELS points accounted by option *i*, *T* is the summed points value of all ELS options concerned and *tHQ*_*i*_ is the percentage of total HQ of all options represented by each option.

For each model the total ELS points and number of units for each option were recalculated to compare with the baseline. Once the ELS composition of each model was calculated the total number of units for each option in each model and the baseline were then multiplied by the average per annum costs per unit (See Table 7 in Appendix) using the costs from the SAFFIE (2007) and Nix (2010), following the establishment and management guidelines laid out in each option (Natural England 2010). Many options had low or no cost. In the case of options requiring sown mixes of plants, the average sowing cost per hectare using a range of mixes from various suppliers was used (See Table 8 in Appendix), however mixes with a total cost of >£1,000/ha, most of which are designed for advanced habitat restoration, were excluded to prevent skewing. The ratio of total costs of implementing all options against total ELS payments (a measure of farmer benefit) were calculated as farmer cost:benefit. The total units of each option were multiplied by their respective HQ score to produce an abstract quantitative measure of the overall benefits of the final option mix for pollinator habitat. As pollination services are largely a public good, each models benefit scores can then be compared with the total ELS payments to gain a measure of public cost:benefit.

**Table 7:** Estimated costs of implementing ELS options

ELS option	Establishment costs	Establishment regularity	Maintenance costs	Maintenance regularity	Average annual cost	ELS Points
EB1/EB2^a^	£0	NA	£0.06	3	£0.04	0.18
EB3	£0	NA	£0.06	2	£0.02	0.42
EB6	£0	NA	£0.44	3	£0.10	0.24
EB7	£0	NA	£0.44	2.5	£0.10	0.08
EB8/EB9^a^	£0	NA	£0.5	3	£0.26	0.34
EB10	£0	NA	£0.5	3	£0.30	0.56
EB12/13^a^	£0	NA	£0.78	1	£0.16	0.11
EC1	£0	NA	£0	NA	£0	16
EC2	£0	NA	£0	NA	£0	11
EC3	£0	NA	£0.78	1	£0.16	0.04
EC4	£963.54	1	£208.75	2	£276.85	380
EC23	£4.54	1	£0	NA	£0.91	1
EC24	£262.49	1	£40.2	2.5	£68.52	400
EC25	£262.49	1	£40.2	2.5	£68.52	400
EE1/EE2^a^	£262.49	1	£26.5	2	£59.02	383.71
EE3	£262.49	1	£26.5	3	£64.32	400
EE4/EE5/EE6^a^	£262.49	1	£26.5	3	£64.32	381.34
EE7	£262.49	1	£26.5	1	£53.72	400
EE8	£262.49	1	£26.5	1	£53.72	400
EF1	£85.64	1	£26.5	3	£24.02	400
EF2	£766.70	3	£40.2	2	£463.86	450
EF4	£303.88	2	£40.2	1	£121.43	450
EF6	£0	NA	£0	NA	£0	120
EF7	£237.99	1	£40.2	2	£63.32	580
EF8	£0	NA	£0	NA	£0	5
EF9	£0	NA	£0	NA	£0	100
EF10	£0	NA	£0	NA	£0	330
EF11	£60.76	1	£0	NA	£10.50	400
EF13	£262.49	5	£40.2	5	£282.29	360
EF15	£0	NA	£0	NA	£0	195
EF22	£0	NA	£0	NA	£0	410
EG1	£281	1	£0	NA	£53.12	200
EG4	£0	NA	£0	NA	£0.00	230
EK1	£85.64	1	£26.5	1	£13.54	400
EK2	£0	NA	£26.5	1	£26.20	85
EK3	£0	NA	£26.5	1	£26.20	150
EK4	£0	NA	£26.5	1	£26.20	150

*Key* *Establishment costs* costs of a single establishment of a unit of the option, where applicable, *Establishment regularity* the number of years out of 5, within a new 5 year contract, which the option must be established, *Maintenance costs* costs of a single years maintenance of a unit of the option, where applicable, *Maintenance regularity* the number of years out of 5, within a new 5 year contract, which maintenance must be undertaken, where applicable, *Average annual cost* the average cost per year of establishing and maintaining one unit of the option over a 5 year contract, *ELS Points* the number of ELS points generated by one unit of the option

^a^Costs and points for these groups of options represent a weighted average of all relevant options

**Table 8:** Number of species of various plant families occurring within ELS applicable seed mixes

Seed mix	1	2	3	4	5	6	7	8	9	10	11	12	13	14	15	16	17	18	19	Total
Apiaceae*	0	0	1	0	0	1	2	0	3	0	0	0	0	1	1	0	0	0	0	6
Asteraceae*	0	1	3	3	3	2	3	2	2	0	0	0	0	6	6	3	0	1	0	12
Boraginaceae*	0	0	0	0	0	0	0	0	2	0	1	0	0	0	0	0	0	0	2	3
Brassicaceae	0	0	0	0	0	0	0	1	2	0	0	0	0	0	0	0	0	0	0	2
Caryophyllaceae	0	0	0	1	1	1	1	1	1	0	2	0	0	1	1	0	0	0	0	9
Dipsacaceae*	0	0	1	0	0	0	0	0	0	0	0	0	0	0	0	0	0	0	0	1
Fabaceae*	6	4	0	1	1	1	1	5	1	5	9	5	5	10	6	2	5	6	2	18
Lamiaceae*	0	0	1	1	1	0	1	1	0	0	0	0	0	0	0	0	0	0	0	5
Lamiales	0	0	0	0	0	0	0	0	1	0	0	0	0	0	0	0	0	0	0	1
Malvaceae	0	1	0	0	0	0	0	0	0	0	0	0	0	0	0	0	0	0	0	1
Plantaginaceae	0	0	0	1	1	1	1	0	0	0	0	0	0	1	1	1	0	0	0	7
Poaceae	7	0	6	6	6	5	4	0	0	0	0	7	6	7	7	7	4	0	0	12
Polygonaceae	0	0	1	0	0	0	1	0	0	0	0	0	0	1	1	1	0	0	0	5
Primulaceae	0	0	0	0	0	0	0	0	0	0	0	0	0	1	1	1	0	0	0	3
Ranunculaceae	0	0	1	1	1	0	1	0	0	0	0	0	0	2	2	1	0	0	0	7
Rosaceae	0	0	0	0	0	0	0	0	1	0	0	0	0	0	0	0	0	0	0	1
Rubiaceae	0	0	1	1	1	1	2	0	0	0	0	0	0	2	2	1	0	0	0	8
Scrophulariaceae	0	0	0	0	0	0	0	0	2	0	0	0	0	0	0	0	0	0	0	1
Violaceae	0	0	0	0	0	0	0	1	0	0	0	0	0	0	0	0	0	0	0	1
Total families	2	3	8	8	8	7	10	6	9	1	3	2	2	10	10	8	2	2	2	5.42
Key families	1	2	4	3	3	3	4	3	4	1	2	1	1	3	3	2	1	2	2	2.37
Total species	13	6	15	15	15	12	17	11	15	5	12	12	11	32	28	17	9	7	4	13.5
Species/family	6.5	2	1.9	1.9	1.9	1.7	1.7	1.8	1.6	5	4	6	5.5	3.2	2.8	2.1	4.5	3.5	2	3.14
Key sp	6	5	6	5	5	4	7	8	8	5	10	5	5	17	13	5	5	7	4	
Cost/Ha	89	134.0	278.4	200	224.8	500	500	2250	1050	105	155	140	70	128	476	436	104	38.8	140.3	
£/family	44.5	44.7	34.8	25	28.1	71.4	50	375	116.7	105	51.7	70	35	12.8	47.6	54.5	52	19.4	70.14	
£/key	89	67.0	69.6	66.7	74.9	166.7	125	750	262.5	105	77.5	140	70	42.7	158.7	218	104	19.4	70.14	
£/sp	6.9	22.3	18.6	13.3	14.9	41.7	29.4	204.6	70	21	12.9	11.7	6.4	4	17	25.7	11.6	5.54	35.07	
£/key sp	14.8	26.8	46.4	40	44.9	125	71.4	281.3	131.3	21	15.5	28	14	7.53	36.62	87.2	20.8	5.54	35.07	

* Key family for promoting pollinators, as identified during the expert survey

Seed mixes are kept anonymous to avoid presenting a commercial advantage to particular manufacturers

### Sensitivity

As with all models utilising expert opinion, there are a number of ways the values used in this study can be biased; foremost, individual expert uncertainty and overconfidence can cause substantial skewing of the results towards certain options. Therefore each model was recalculated by Jackknifing, removing one expert each time before calculating the PHB. The percentage difference in total farmer costs between each Jackknife and the average of all Jackknives was then compared with the version for all experts. Strong effects from this deletion compared with the “all experts model” would indicate that the model is biased by highly polarised expert opinions. Similarly, expert reported confidence may not be a reliable means of weighting the PHB scores—therefore each model was recalculated using unweighted PHB scores to determine the percentage change caused by weighting. Strong changes would indicate that the weighting system creates an inherent bias. Finally, it is possible that using expert opinion to weight ELS points may not produce an option mix which is substantially different from developing a model based on ELS points alone. Consequently each model was recalculated using only ELS points to estimate relative PHB. Strong differences would indicate that the expert weighting has a substantial impact in guiding the redistributions over ELS points alone.

## Results

### ELS habitat quality scores

Of the 35 experts contacted, 27 (77 %) responded; eighteen of which (51 %) returned completed questionnaires while nine (25 %) declined to participate due to concerns with the use of expert questionnaires to inform ecological models, concerns over their own expertise or a lack of time available. As expected, option EF4 (Nectar flower mix) was given the greatest PHB with a mode score of 3 and a mean of 2.83 (Table 2). On average, each expert allocated six options a PHB score of 0 and an average of 1.5 options a PHB score of 3. Expert confidence in responses was generally high with 13 (72 %) giving confidence scores of 3 or 4 and only two (11 %) experts giving scores of 1. When weighted for expert confidence, mean PHB values for all options fell sharply (mean 0.86); EF4 remained the highest rated (PHB 2.83) followed by options for hedges EB10, EB3, EB8/9 and woodland edges EC4 (mean PHB ≥ 1.75) while options for winter stubbles EF6, EF22 and EG4 remained the lowest rated options (mean PHB ≤ 0.5).

### Model costs and benefits

The three most important options in the 2012 baseline option mix were for hedges and low input grassland EB1/2, EK2 and EK3 (Table 2) which collectively account for 50 % of total points. The grassland option area was 216 % greater than the arable option area, most likely because of high uptake of these options in less productive areas (Hodge and Reader 2010). Total costs of the ELS options considered from a 2012 baseline were estimated at £32.2 M, giving a £1:£4.13 cost:benefit ratio compared with the ELS payments (£133 M) provided. In terms of pollinator habitat quality; the baseline ELS provides 200 M units total HQ benefit, quantitatively equivalent to 1.5 units of HQ per £1 of ELS payment. The most costly options were those that included seed costs (See Table 7 in Appendix). EB1/2, EF6, EK2 and EC2 contributed the greatest proportion of points to the hedge/ditch (48.1 %), arable (18 %), grassland (18.6 %) and plot/tree (75.5 %) option categories respectively.

To assess the costs of providing pollinator habitat oriented ELS compositions, the study utilised expert opinion to weight three redistributions of ELS options by multiplying the PHB values provided by the ELS points conferred to each option. The most beneficial options in each category were EB10 (hedge/ditch option), EF4 (arable option), EK1 (grassland option) and EC1 (tree/plot option). Under Model A the number of units within each of the four option categories was restructured to reflect the benefits to pollinator habitat, increasing the quality of the absolute area currently managed (Table 3). This increased the area managed under ELS by 108.3 % (Table 4) but also produces the greatest total private costs (~£59.1 M) and more than doubles both public costs (£144 M; 108 %) and total HQ benefits (+140 %). This model therefore results in the smallest loss of private cost:benefit and the greatest public cost:benefit gain.

Model B re-allocates ELS points within each option category to maintain current ELS expenditure but allows option area to vary. This produces substantial declines in the total number of units across most option categories, particularly grassland options which contracts by 64 % (Table 5). Overall, option costs rise by £16.6 M, however as ELS payments remain constant, this reduces cost:benefit ratio by 34 % to £1:£2.73. By contrast the cost:benefit to the public rises by almost as much as the more expensive Model A, although total HQ benefits only rise by 14 %.

Model C restructures option composition more radically by reallocating ELS points between all options regardless of category. This model results in substantial reductions in both hedge/ditch and grassland options but increases the number of arable and tree per plot based units. Total annual costs of options under this model rises by £12.4 M, reducing cost:benefit to farmers by 28 % to £1:£2.98. This model also produces the lowest gains in HQ benefits and public cost:benefit ratio (7 %). Under all three models, option EK2 (low input grassland), one of the most significant options under the baseline scenario, declines by ≥93 % (≥269,486 ha) while options EB10 (combined hedge and ditch management), options EC4 (maintain woodland edge) become the most widespread under all three variations and EF4 (nectar flower mix) rises in area by 480 % (Models A and C) and 260 % (Model B) under all models (Table 3).

### Sensitivity

To assess the sensitivity of models to factors which may distort the estimates, each model was subject to three re-analyses. First, to assess the sensitivity of the model to individual respondents, the PHB values were recalculated 18 times with one respondent deleted from one of the iterations and compared with the original “all experts” group. All three models were largely uninfluenced by individual respondents; removing any individual respondent produced recalculated costs and ELS points between ±1 % of the original estimates in any model and the difference between the mean costs across all expert models (Table 6) and the original estimates (Table 4) were negligible (<0.1 %) under all three models. In Models A and B, the total HQ benefit remained within ± 1 % of the all expert models when any individual expert was removed, reflecting a strong consensus among experts. Under Model C, however, these benefits ranged from −4 to +7.5 % (average 1.2 %) of the original estimates, due to the stronger influence of differences in option PHB values have on overall option composition.

A second sensitivity analysis evaluated the impact of expert confidence weighting on the model outcome by instead using unweighted average PHB. Results indicate that respondent weighting had a relatively small effect upon the total costs estimated; changing by <0.5 % of their original values (Table 6). Overall, the importance of each option to their categorical total PHB remained stable at ±0.64 % of the original values, although substantial differences in total units arise for some options due to the greater differences between PHB values. Notably, due to the lower PHB values for several other options, EF4 (nectar flower mix) had a greater coverage in all three unweighted models. Changes in the total units of option categories in Model B and total ELS costs of Model A were negligible (<5 %) compared to the weighted PHB analysis. Model C however produces 38 % less tree/plot option units while the area of arable options area grows by 23 % more than the unweighted model. Due to the high degree of agreement between experts as to the most beneficial options, the unweighted models produced <2 % lower total HQ benefit than the weighted models.

A third re-analysis assessed the effects of PHB model outcomes compared with ELS points alone. In Model A this results in a substantially smaller increase of several high PHB value options, notably EB10 (combined hedge and ditch management), EC4 (management of woodland edges) and EF4 (nectar flower mix). In Model B, without the weighting effect of expert opinions, options within each category occupied an identical number of units to all other options within the category. This is an effect of the habitat quality metric in the formula; the pHQ of an individual option now represents the proportion of sum ELS points within the category it represents; 24.6 M metres (hedge/ditch), 23,466 ha (grassland), 6,475 ha (arable) and 68,186 units of each plot/tree based item. More extreme trends occur in Model C as all options now occupy the same number of units scaled to the magnitude of their ELS points; 13.2 M metres (hedge/ditch), 13,268 ha (arable and grassland) and 132,685 units of each plot/tree based option. Producer costs of Models A and C were 9 % lower (Table 5) due to the reduced uptake of high cost, high PHB options reducing total PHB by 31–41 % compared with the expert weighted option distribution and 4–36 % less than the baseline.

## Discussion

### Habitat benefits of ELS options

Using a panel of 18 experts, this study estimated the potential of options in England’s entry level stewardship (ELS) to provide good quality habitat for pollinators on a simple 0–3 scale. Expert patterns generally showed agreement with past research, with many of the most highly rated options having significant empirical backing. In particular UK field studies (e.g. Pywell et al. 2011; Potts et al. 2009; Lye et al. 2009) and international meta-analyses (Batary et al. 2010; Scheper et al. 2013) have demonstrated the benefits of Nectar flower mixes (EF4), field margins (EE1-6) and low inputs grasslands (EK3) on wild pollinator abundance and diversity. However, expert consensus did not always match published literature. For instance, although Lye et al. (2009) indicated that hedgerows were less attractive to emergent bumblebee queens than grass margins; Hedgerow Management options (EB1-3) were among the highest rated options. Similarly Potts et al. (2009) demonstrated benefits to bumblebee abundance from management similar to EG1 (under sown spring cereals) however expert pollinator habitat benefit (PHB—Eq. 1) score was low for this option. These trends may stem from the broader taxonomic scope of the panel than previous studies. For many options however, expert opinion has little or no direct empirical backing. In particular options EB8-10 (combined hedge and ditch management), and EC24/25 (Hedgerow tree buffer strips on cultivated/grassland), have no direct studies for the benefits to pollinators but are likely to provide high quality nesting resources for a broad range of species on otherwise crop/grass dominated land.

While lacking the rigors of primary ecological research, this study demonstrates that expert opinion can be used to provide an insight into the benefits of options within ELS to specific taxa and ecosystem services. Indeed many of the highest rated options in this study are now recommended for improving habitat for pollinators in the current, 4th edition of the ELS handbook (Natural England 2013b). However, the range of possible values of PHB that experts were able to give may impact upon the habitat quality (HQ—Eq. 2) values and subsequent analysis by making the differences in benefits between options more coarse. Furthermore this also assumes no variation in quality of option implementation either by management, or by spatial (proximity to source habitat) or temporal factors (succession), preventing a more accurate estimate of long term benefits within landscapes. Altering the scale of response (e.g. to a continuous 0–1 scale) to better emphasise differences in benefits between options may allow more precise quality appraisals. Alternatively, experts could give confidence intervals along the same scales to represent variation in option management or synergies with other options.

### Costs and benefits of model applications

Using three models, PHB scores were translated into new compositions of options based on a 2012 baseline. The total costs of restructuring ELS towards a composition reflecting the benefits to pollinators were then estimated, using prior data, at £91.4–£44.8 M. This increase of £53.9–£12.4 M over the baseline (£32.2 M) reduces the benefits of ELS payments to farmers relative to their costs by up to 52 %. Nonetheless, these private costs are substantially below the estimated value of crop production added by pollination services (£430 M—Smith et al. 2011). If the value of ELS payments is added, representing society’s expenditure on incentivising these options, total costs are estimated at £308.7–£162.5 M, with private costs rising at a faster rate than public benefits. The benefits of these options mixes, in terms of total quantitative habitat quality scores, varied strongly between models but all three result in an increase in overall habitat quality. Notably, although restructuring the current area of ELS options to redistribute existing area use (Model A) more than doubles the total habitat quality provided by ELS, it requires almost all English farmland (9.3 M ha; DEFRA 2013) to be enrolled in the scheme and provides negligible public benefits over a redistribution based on current ELS expenditure (Model B). Subsequently, this study demonstrates that the benefits of ELS to pollinator habitats can be greatly enhanced without additional public expense by encouraging existing participants to switch options.

Although based upon previous establishment and maintenance cost estimates (Nix 2010; SAFFIE 2007), these values do not account for variation in costs that may arise, such as variations in seeding costs with optimised mixes tailored to local floral diversity or service delivery or for specific successional management. Furthermore these costs do not include opportunity costs in placing ELS options on productive land, production losses resulting from extensified production and pest encroachment (e.g. Carvell 2002) or the impact of reduced production on consumer prices. Such opportunity costs could potentially be captured with proxies such as the per hectare profit of key arable crops, grazing livestock or intensive milk production, potentially resulting in a net gain from added production value if land is brought back into production (models B and C). However, as ELS options are often applied to land with low or unreliable productivity and variation in production costs between different regions, these opportunity costs would likely be exaggerated. Legislative regulation such as the Hedgerows Act 1997 (HM Government 1997) also restrict land owners ability to take advantage of particular opportunity costs, making them largely inappropriate for some options. Furthermore, many options also provide uncaptured economic benefits such as increased soil quality and erosion control, profit from placing ELS options on unproductive land and reduced risk of environmental contamination (Wratten et al. 2012). Therefore, while the costs of conservation through ELS may be substantial, the economic value of ecosystem service benefits provided are likely to be substantially greater. Future studies could readily expand on this methodology to develop optimisation models to maximise the benefits of ELS to a wider range of taxa and ecosystem services.

Sensitivity analyses demonstrated that final option mixes of the three models were not biased by either the weighting of expert PHB scores or the influence of individual experts. Differences in total costs between weighted and unweighted models stem from the altered distributions of some options when all experts opinions are considered equal as the differences between PHB values becomes greater. However, most experts were equally confident, this effect is small. The minor influence of individual experts reflects a strong consensus among experts on many options and the effectiveness of averaging in reducing between expert bias (Czmebor et al. 2011). Differences in composition are greatest when PHB values are not used to weight ELS points, indicating the significant influence of expert weighting for specific taxa rather than using more general biodiversity value alone. As such, the option compositions produced may have lower or negative benefits on other taxa; for example cereal headlands for birds (option EF9) have a very low PHB score.

While coverage of higher PHB options increased under all models, option redistribution may result in quality habitat becoming more dispersed throughout the landscape; Models B and C by reduction of absolute AES coverage and Model A by the increased points value of the scheme broadening distribution of existing units. Furthermore, the models used to estimate these redistributions are based heavily upon the assumption that the existing area encompassed by ELS is adequate. Although experts were asked what percentage of UK farmland they believe should contain good quality pollinator habitat to halt or reverse pollinator declines only 78 % of respondents completed these questions, all indicated no or little confidence in their answers. Other respondents refused to answer, citing concerns over the implications of such answers. Subsequently, the methods presented are appropriate for estimating the costs of pollinator habitat conservation with current knowledge.

### Enhancing ELS impacts

While many ELS options can provide good quality habitat for pollinators, it is highly unlikely that these measures alone would be able to sustain diverse pollinator communities and are best employed in moderately diverse landscapes, where remnant source populations exist in pockets of high quality semi-natural habitats (Scheper et al. 2013; Batary et al. 2010). By linking and diversifying these semi-natural habitats, ELS options could potentially provide significant value added to the overall landscape (Garibaldi et al. 2011; Ricketts and Lonsdorf 2013). However, these habitat patches may be widely dispersed across the landscape and be owned by a number of stakeholders with different objectives. To date there are no specific incentives for farmer co-operation within ELS and beyond ELS (e.g. the higher level stewardship—Natural England 2013c) and, aside from habitats protected by the EU’s Habitats Directive (e.g. hay meadows), few incentives for producers to maintain semi-natural habitats outside of already high diversity areas.

Unfortunately, because most ELS option uptake is opportunistic, often where measures are already implemented (Sutherland 2009) or where production is low enough that payments are profitable (Hodge and Reader 2010), the uptake of many of the ELS options most beneficial to pollinators remains limited. For example, although uptake of EF4 has increased >100 % since 2007, this still only represents ~1 % of ELS expenditure (Hodge and Reader 2010; Cloither 2013). However, World Trade Organisation “green box” guidelines prevent payments being made in excess of projected costs (WTO 1995) limiting the capacity for expanded financial incentives. This could be rectified by making options more flexible, as seen in recent revisions allowing EF4 (nectar flower mix) to be integrated into crop rotations (Natural England 2013b), and illustrating the broader ecosystem service benefits of many options (Wratten et al. 2012).

Beyond economic considerations, sociological incentives, such as the government endorsed campaign for the farmed environment (CFE) aim to increase uptake of the most environmentally beneficial options. However the CFE has a broad scope prioritising >60 % (42) of 2010 ELS options (Cloither 2013) and farmer decisions regarding AES are thought to be largely insensitive to the opinions of peers (“social norms”—Sutherland 2009), calling the effectiveness of social incentives into question. Burton et al. (2008) further suggest that AES uptake may be limited by the lack of associated cultural capital, a measure of accomplishment associated with land management that can be compared over years and between land holders. Presently, ELS options are simply applied without specific rewards or prestige for the ecological quality of their application or outcomes; consequently, encouraging an emphasis on overt quality elements (e.g. high floral diversity) or outcomes (e.g. increases in iconic species) could improve the social impetus to uptake these options.

Finally, several members of the expert panel emphasised the need for a more detailed monitoring scheme for insect pollinators in the UK in order to assess the overall effectiveness of different interventions on pollinator numbers. Although the costs of such a scheme, able to detect changes in pollinator abundance and diversity, would be ~£263,000/year (over 5 years) (Lebuhn et al. 2013) the data produced would be highly valuable to optimising ELS effectiveness and providing measures of success for use in cultural capital (Burton et al. 2008) or payments for ecosystem services schemes (Farley and Costanza 2010).

## Conclusions

Using an expert panel to inform a redistribution of ELS options, this study indicates that England’s entry level stewardship has the potential to provide substantial benefits to pollinator habitat, however these options are not yet widely adopted. The use of expert panels allowed a more comprehensive assessment of the benefits of options than current literature alone. Private costs incurred in altering the composition of ELS options towards one that reflects the relative benefits of each option to pollinator habitat are estimated as £59.3–£12.4 M. The models used in this study demonstrate the potential for management options in ELS to significantly increase the overall quality of habitat for pollinators without additional public expenditure or private land use, simply by participants switching options. This study highlights the need to consider the costs and benefits of specific ELS options rather than the scheme as a whole if the scheme is to provide effective conservation without compromising flexibility or increasing annual costs. Future research should aim to identify means of further incentivising participants to employ the most beneficial options.

## Appendix

See Tables 7 and 8.

## References

[CR1] Batary P, Baldi A, Sarospataki M, Kohler F, Verhulst J, Knop E, Herzog F, Kleijn D (2010). Effect of conservation management on bees and insect-pollinated grassland plant communities in three European countries. Agric Ecosyst Environ.

[CR3] Burgess PJ, Morris J (2009). Agricultural technology and land use futures: the UK case. Land Use Policy.

[CR4] Burton RJ, Kuczera C, Schwarz G (2008). Exploring farmers’ cultural resistance to voluntary agri-environmental schemes. Sociol Ruralis.

[CR6] Carvalheiro LG, Kunin WG, Keil P, Aguirre-Gutierrez J, Ellis WE, Fox R, Groom Q, Hennekens S, Landuyt W, Meas D, de Meutter FV, Michez D, Rasmont P, Ode B, Potts SG, Reemer M, Roberts SPM, Schaminee J, WallisDeVires MF, Biesmeijer JC (2013). Species richness declines and biotic homogenisation have slowed down for NW-European pollinators and plants. Ecol Lett.

[CR7] Carvell C (2002). Habitat use and conservation of bumblebees (*Bombus* spp.) under different grassland management regimes. Biol Conserv.

[CR8] Carvell C, Meek WR, Pywell RF, Goulson D, Nowakowski M (2007). Comparing the efficacy of agri-environment schemes to enhance bumble bee abundance and diversity on arable field margins. J Appl Ecol.

[CR10] Cloither L. (2013) Campaign for the farmed environment: entry level stewardship option uptake. https://www.gov.uk/government/uploads/system/uploads/attachment_data/file/183937/defra-stats-foodfarm-environ-obs-research-setaside-farmenviroment-ELSinCFEjan13-130214.pdf

[CR11] Cook DC, Thomas MB, Cunningham SA, Anderson DL, de Barro PJ (2007). Predicting the economic impact of an invasive species on an ecosystem service. Ecol Appl.

[CR13] Czmebor C, Morris WK, Wintle BA, Vesk PA (2011). Quantifying variance components in ecological models based on expert opinion. J Appl Ecol.

[CR14] DEFRA (2011) Biodiversity 2020: a strategy for England’s wildlife and ecosystem services. http://www.defra.gov.uk/publications/files/pb13583-biodiversity-strategy-2020-111111.pdf

[CR15] DEFRA (2013) Agriculture in the United Kingdom. https://www.gov.uk/government/statistical-data-sets/agriculture-in-the-united-kingdom last updated 25/06/13

[CR17] Dicks LV, Showler DA, Sutherland WJ (2010) Bee conservation: evidence for interventions www.conservationevidence.com

[CR18] European Commission (2013) Memo: CAP reform—an explanation of the main elements. http://europa.eu/rapid/press-release_MEMO-13-621_en.htm

[CR19] Farley J, Costanza R (2010). Payments for ecosystem services: from local to global. Ecol Econ.

[CR20] Forup ML, Memmott J (2005). The restoration of plant–pollinator interactions in hay meadows. Restor Ecol.

[CR22] Garibaldi LA, Aizen MA, Klein AM, Cunningham SA, Harder LD (2011). Global growth and stability of agricultural yield decrease with pollinator dependence. Proc Natl Acad Sci USA.

[CR23] Garibaldi (2013). Wild pollinators enhance fruit set of crops regardless of honey–bee abundance. Science.

[CR24] Gill RJ, Ramos-Rodrigez O, Raine NE (2012). Combined pesticide exposure severely affects individual- and colony-level traits in bees. Nature.

[CR25] HM Government (1997) The hedgerow regulations 1997. http://www.legislation.gov.uk/uksi/1997/1160/contents/made

[CR26] Hatfield RG, LeBuhn G (2007). Patch and landscape factors shape community assemblage of bumble bees, *Bombus* spp. (Hymenoptera: Apidae), in montane meadows. Biol Conserv.

[CR27] Henry M, Beguin M, Requier F, Rollin O, Odoux J-F, Aupinel P, Aptel J, Tchamitchian S, Decourtye A (2012). A common pesticide decreases foraging success and survival in honey bees. Science.

[CR28] Hodge I, Reader M (2010). The introduction of entry level stewardship in England: extension or dilution in agri-environment policy?. Land Use Policy.

[CR29] Isbell F, Tilman D, Polasky S, Binder S, Hawthorne P (2013). Low biodiversity state persists two decades after cessation of nutrient enrichment. Ecol Lett.

[CR30] Kleijn D, Sutherland W (2003). How effective are European agri-environmental schemes in conserving and promoting biodiversity?. J Appl Ecol.

[CR31] Kleijn D, Baquero RA, Clough Y, Diaz M, de Esteban J, Fernandez F, Gabriel D, Herzog F, Holzschuh A, Johl R, Knop E, Kruess A, Marshall EJP, Steffan-Dewenter I, Tscharntke T, Verhulst J, West TM, Yela JL (2006). Mixed biodiversity benefits of agri-environment schemes in five European countries. Ecol Lett.

[CR33] Klein AM, Vaissiere BE, Cane JH, Steffan-Dewenter I, Cunningham SA, Kremen C, Tscharntke T (2007). Importance of pollinators in changing landscapes for world crops. Proc Roy Soc Lond B.

[CR34] Kuldna P, Peterson K, Poltimaee H, Luig J (2009). An application of DPSIR framework to identify issues of pollinator loss. Ecol Econ.

[CR36] LeBuhn G, Droege S, Connor EF, Gemmill-Herren B, Potts SG, Minckley RL, Griswold T, Jean R, Kula E, Roubik DW, Cane J, Wright KW, Frankie G, Parker F (2013). Detecting insect pollinator declines on regional and global scales. Conserv Biol.

[CR37] Lonsdorf E, Kremen C, Ricketts T, Winfree R, Williams S, Greenleaf S (2009). Modelling pollination services across agricultural landscapes. Ann Bot.

[CR38] Lye G, Park K, Osborne J, Holland J, Goulson D (2009). Assessing the value of rural stewardship schemes for providing foraging resources and nesting habitat for bumblebee queens (Hymenoptera: Apidae). Biol Conserv.

[CR40] Natural England (2010) entry level stewardship, 3rd edition. http://naturalengland.etraderstores.com/NaturalEnglandShop/NE226

[CR41] Natural England (2012) New (O)ELS options available and option changes from 1st January 2013. http://www.naturalengland.org.uk/Images/new-ELS-options-info-note_tcm6-32527.pdf

[CR42] Natural England (2013a) Land Management Update 11: May 2013. http://www.naturalengland.gov.uk/Images/lmupdate11_tcm6-35842.pdf

[CR44] Natural England (2013b) Entry Level Stewardship, 4th Edition. http://publications.naturalengland.org.uk/file/2781958

[CR43] Natural England (2013c) Higher Level Stewardship, 4th Edition. http://publications.naturalengland.org.uk/file/2819648

[CR45] Nix J (2010). Farm management pocketbook.

[CR46] Ollerton J, Winfree R, Tarrant S (2011). How many flowering plants are pollinated by animals?. Oikos.

[CR47] Potts SG, Woodcock BA, Roberts SPM, Tscheulin T, Pilgrim ES, Brown VK, Tallowin JR (2009). Enhancing pollinator biodiversity in intensive grasslands. J Appl Ecol.

[CR48] Potts SG, Roberts SPM, Dean R, Marris G, Brown MA, Jones R, Neumann P, Settele J (2010). Declines of managed honeybees and beekeepers in Europe. J Apic Res.

[CR49] Pywell RF, Meek WR, Loxton RG, Nowakowski M, Carvell C, Woodcock BA (2011). Ecological restoration on farmland can drive beneficial functional responses in plant and invertebrate communities. Agric Ecosyst Environ.

[CR59] Ricketts T, Lonsdorf E (2013) Mapping the margin: comparing marginal values of tropical forest remnants for pollination services. Ecol Appl 25:1113–1123 10.1890/12-1600.1 23967579

[CR50] SAFFIE (2007) Cost:benefit analysis of the best practices for increased biodiversity, Chapter 8, HGCA. http://www.hgca.com/publications/documents/cropresearch/PR416_SAFFIE_8_Cost_benefit_analysis.pdf

[CR52] Scheper J, Holzschuh A, Kuussaari M, Potts SG, Rundlöf M, Smith HG, Kleijn D (2013). Environmental factors driving the effectiveness of European agri-environmental measures in mitigating pollinator loss—a meta-analysis. Ecol. Lett.

[CR53] Smith P, Ashmore M, Black H, Burgess P, Evans C, Hails R (2011). UK national ecosystem assessment, chapter 14: regulating services.

[CR54] Stoate C, Baldi A, Beja P, Boatman ND, Herzon I, van Doorn A, de Snoo GR, Rakosy L, Ramwell C (2009). Ecological impacts of early 21st century agricultural change in Europe. J Environ Manag.

[CR55] Sutherland L-A (2009). Environmental grants and regulations in strategic farm business decision-making: a case study of attitudinal behaviour in Scotland. Land Use Policy.

[CR56] Vanbergen A, The Insect Pollinators Initiative (2013). Threats to an ecosystem service: pressures on pollinators. Front Ecol Environ.

[CR57] World Trade Organisation (1995) Agreement on Agriculture. http://www.wto.org/english/docs_e/legal_e/14-ag.pdf

[CR58] Wratten SD, Gillespie M, Decourtye A, Mader E, Desneux N (2012). Pollinator habitat enhancement: benefits to other ecosystem services. Agric Ecosyst Environ.

